# The emergence of neuroinvasive *Cryptococcus:* why eucalyptus-rich regions, especially in Africa, may be facing greater risk

**DOI:** 10.3389/fcimb.2025.1727496

**Published:** 2026-03-02

**Authors:** Hilaire Irere, Racheal Dangarembizi, Liliane Mukaremera, Ivy M. Dambuza

**Affiliations:** 1 Medical Research Council Centre for Medical Mycology at the University of Exeter, Department of Biosciences, Faculty of Health and Life Sciences, Exeter, United Kingdom; 2 Division of Physiological Sciences, Department of Human Biology, Faculty of Health Sciences, University of Cape Town, Cape Town, South Africa; 3 Neuroscience Institute, Faculty of Health Sciences, University of Cape Town, Groote Schuur Hospital, Cape Town, South Africa; 4 Medical Research Council Centre for Medical Mycology Africa Unit, Cape Town, South Africa

**Keywords:** Cryptococcus neoformans, eucalyptus, inositol, neurotropism, virulence

## Abstract

Cryptococcal meningitis (CM) is often approached under the assumption that *Cryptococcus neoformans* strains from any environment have equal potential for rapid central nervous system (CNS) invasion. This one-size-fits-all view shapes both treatment strategies and how virulence is studied. In this short communicationcont, we highlight increasing evidence showing that environmental nutrient profiles can “pre-condition” *C. neoformans* for distinct pathogenic trajectories. For example, phosphate-rich pigeon guano often yields small-capsule morphotypes suited for systemic dissemination, but their propensity for CNS invasion appears modest compared to inositol-rich eucalyptus-associated strains, which adopt small-cell, large-capsule phenotypes with cell surface features optimized for blood-brain barrier traversal and CNS adaptation. While pigeon guano-derived isolates can cause CM under certain host or epidemiological contexts, the scale of CNS disease linked to regions with more eucalyptus trees appears disproportionately higher in affected regions. Recognizing these ecological influences, we suggest a reframing of CM not as an inevitable outcome of exposure, but as a risk also modulated by environmental context, offering new avenues for surveillance, prediction, and targeted intervention.

## Introduction

**Figure 1:**
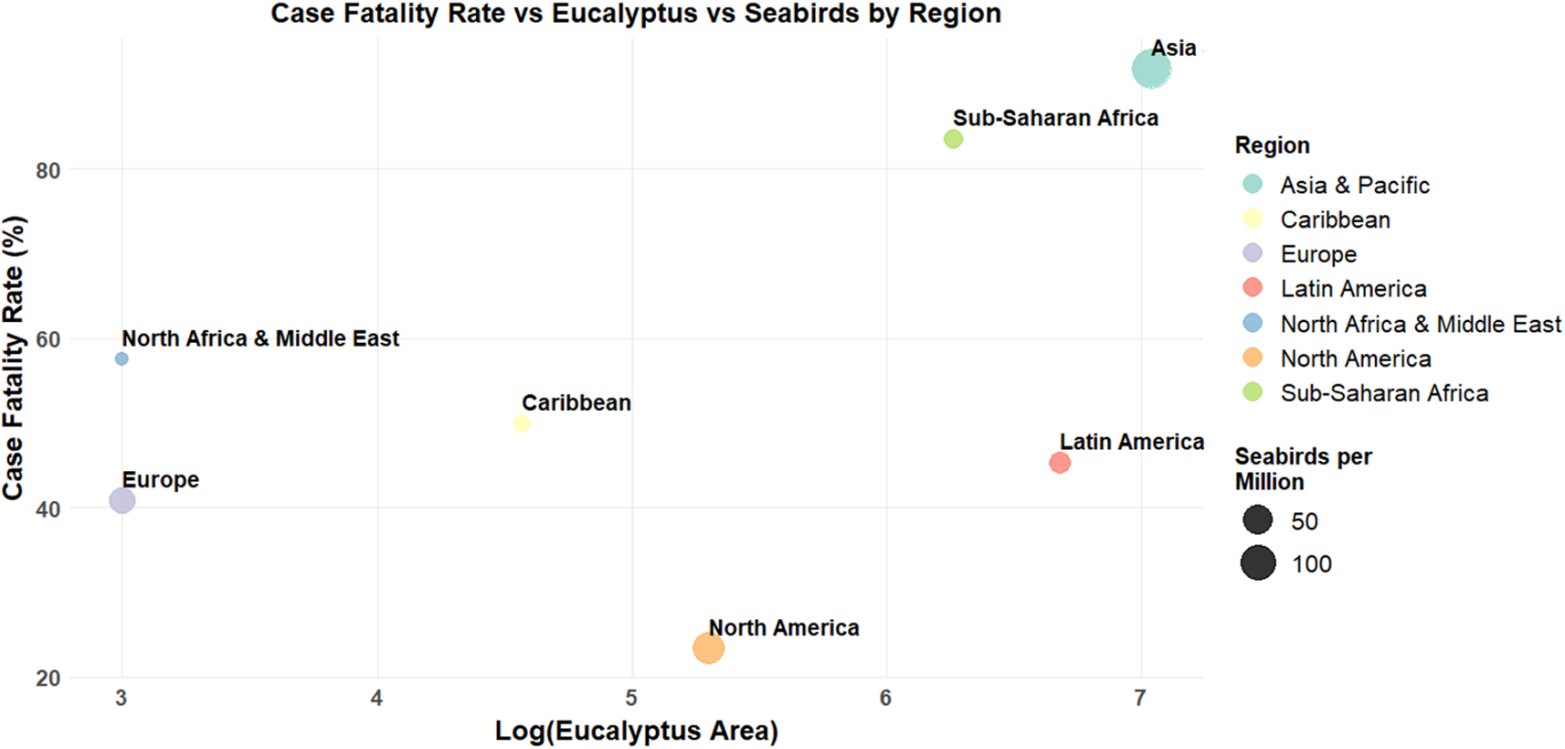
Elevated cryptococcal meningitis fatality rates are associated with regions of extensive eucalyptus coverage. This map integrates reported annual incidence and mortality data for cryptococcal meningitis (CM) with global distribution data for eucalyptus trees and seabird populations. Eucalyptus regions were highlighted due to their high inositol content, which may enhance *Cryptococcus* virulence. Seabirds were included as a proxy for avian-associated *Cryptococcus* reservoirs in lieu of comprehensive global data on pigeon populations, which are primarily available only for Europe and North America (FA0 reports, 2000; Rajasingham et al., 2017; Otero et al., 2018). South America is interesting, despite its extensive eucalyptus distribution, exhibits lower cryptococcal meningitis fatality rates compared to other eucalyptus-dominated regions. This divergence underscores the need for further investigation into regional ecological and epidemiological factors which may be influenced by climate variability and seasonal influence on nutrient profiles in eucalyptus trees, including inositol concentrations (Merchant et al., 2006; Souden et al., 2020).

Cryptococcal meningitis (CM) remains a leading cause of death among people living with HIV/AIDS, with sub-Saharan Africa accounting for nearly 70% of global CM fatalities annually (Rajasingham et al., 2022). While this high burden is often attributed to factors such as immunosuppression and limited access to anti-fungal therapy, growing evidence suggests that the environmental ecology of pathogens also plays a critical role in shaping disease outcomes (Walther and Ewald, 2004; Kebabonye et al., 2023). Here, we propose a framework that also considers the environmental source of *C. neoformans* when approaching research on host adaptation, translational applications, and clinical interventions. The observed regional differences in the global burden and mortality of CM, together with variation in the abundance and composition of *Cryptococcus* environmental niches, lend support to this proposal (**Figure 1**). Among the primary environmental niches of *C. neoformans*, pigeon guano and eucalyptus trees are particularly notable. These reservoirs differ not only in their geographical distribution with pigeon guano prevalent in urban environments and eucalyptus trees dominating peri-domestic and rural landscapes in sub-Saharan Africa and parts of Asia (May et al., 2016; Poplin et al., 2024) (Global data on forest plantations resources), but also in their chemical composition. For instance, pigeon guano is rich in nitrogen and phosphate at the level of 4% and 1%, respectively (Duffaut et al., 2023), whereas eucalyptus bark and leaves contain high concentrations of inositol around 6.5 µg/cm^2^ on the *Eucalyptus camaldulensis* leaf surface (Xue et al., 2007; Xue et al., 2010). Both nutrients are known to modulate key biological processes, including metabolism, mating, virulence factors development, and traits central to the pathogenesis of *C. neoformans* (Nielsen et al., 2007). Together, these nutrient-rich but compositionally distinct reservoirs create environmental heterogeneity that acts as a selective landscape, shaping fungal fitness and virulence potential prior contact with the human host. Importantly, this ecological variation mirrors global patterns in CM incidence and mortality, with the highest burden and case fatality rates reported in sub-Saharan Africa and parts of Asia regions characterized by widespread eucalyptus trees coverage (FA0 reports, 2000; Rajasingham et al., 2017; Rajasingham et al., 2022). In contrast, regions like Europe and North America, where eucalyptus trees are scarce, but pigeon populations are abundant, tend to report significantly lower CM mortality (Rajasingham et al., 2017; Otero et al., 2018; Rajasingham et al., 2022) (**Figure 1**). Regional differences in mortality are also evident between countries within the same geographic area, with variation correlating to the extent of local eucalyptus forest coverage. This pattern is exemplified in East Africa, where Uganda maintains approximately 11, 000 hectares of eucalyptus forest compared to Ethiopia’s 506, 000 hectares (Dessie and Erkossa, 2011). These environmental differences correlate to the markedly different clinical outcomes with Uganda reporting CM mortality rates of 20-42%, while Ethiopia experiences substantially higher rates around 60% (Kambugu et al., 2008; Rakotoarivelo et al., 2020; Ibe et al., 2023; Tufa et al., 2023). Similarly, in high-income settings, Australia has roughly 101 million hectares of eucalyptus forests compared to less than 1.3 million hectares across all Europe (Government Department of Agriculture, A. & Resources, W, 2018). Correspondingly, for example CM mortality in France is reported at approximately 11.5%, whereas Australia’s mortality rate is notably higher at 21.7% (Dromer et al., 2007; van der Torre et al., 2022; Coussement et al., 2023). This geographic correlation suggests that environmental factors, particularly the abundance of eucalyptus trees, may modulate the pathogenicity of *C. neoformans* and influence disease severity in affected populations. Although host-related factors such as immune status, genetics, microbiome composition, diet, and socioeconomic conditions undoubtedly contribute to CM outcomes, they alone do not fully account for these regional disparities (Stewart et al., 2025). This points to a more nuanced framework in which the interaction between pathogen and environmental niche influences disease progression and virulence expression. To understand how environmental conditions might prime *C. neoformans* for virulence it is essential to consider the life cycle of the fungus and the influence of its ecology. Unlike many obligate pathogens, *C. neoformans* is an environmental saprobe that can complete its life cycle and evolve in the environment (Hallas-Møller et al., 2024). This ecological independence enables the pathogen to interact with a range of dead and living organisms in the environment that may shape the expression and maintenance of virulent traits.

Two concepts help explain how such off-host environments can drive pathogenic potential. The sit-and-wait hypothesis proposes that pathogens capable of persisting for extended periods outside a host like *C. neoformans* spores with a dormancy capacity as part of their life cycle are under reduced evolutionary pressure to preserve host viability (Barbosa and Schultz, 1987; Walther and Ewald, 2004). As a result, they can afford to evolve and maintain higher virulence, since transmission and survival do not rely on host survival. Crucially, while these pathogens are in the environment, they actively respond to and are shaped by environmental conditions, including nutrient composition, microbial competitors, and physical stressors. This prolonged environmental exposure with different nutrients may prime *C. neoformans* with traits advantageous for infection. Similarly, the Pharaoh’s curse hypothesis suggests that pathogens with long-lived environmental propagules such as *C. neoformans* spores or desiccated yeast cells may accumulate traits that enhance virulence over time because of extended environmental exposure (Bonhoeffer et al., 1996). *C. neoformans*, which can survive for long periods in nutrient-variable niches exemplifies these principles. In this way, environmental nutrient landscapes do not merely support fungal survival, they actively shape the pathogenic potential of *C. neoformans*. In fact, others have suggested that survival of *C. neoformans* within amoebic predators is thought to select traits that also enhance its ability to resist uptake by mammalian phagocytic cells (Fu et al., 2021), for more detailed review see Arturo Casadevall, 2025 (Casadevall, 2025). This highlights the response of *C. neoformans* to environmental cues to shape future relations with human hosts.

**Figure 2:**
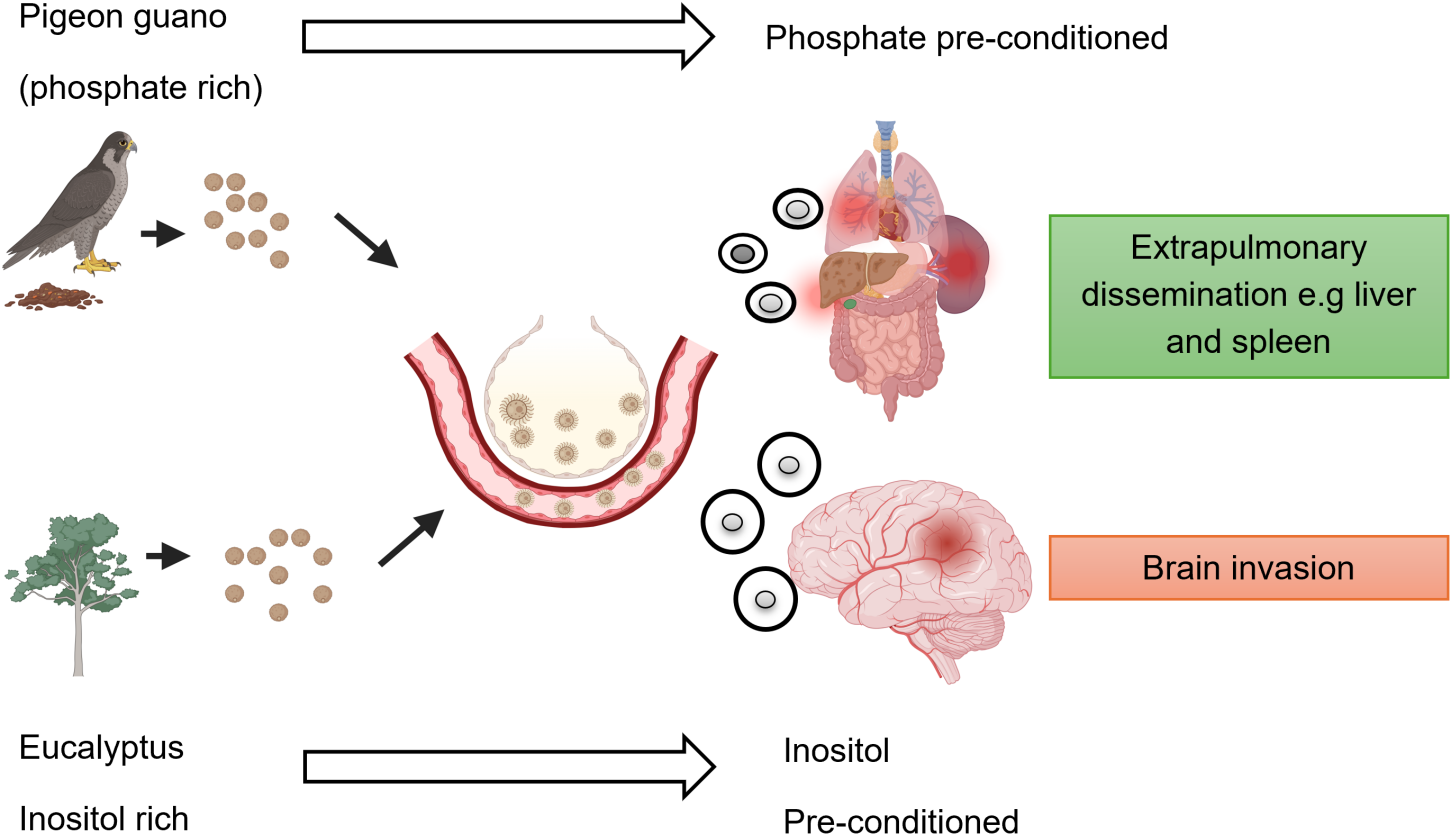
Proposed model on how environmental nutrient composition shapes cryptococcus virulence. This schematic illustrates how distinct environmental reservoirs - pigeon guano (uric acid and phosphate-rich) versus eucalyptus trees (inositol-rich) - influence the morphological differentiation of *Cryptococcus neoformans* and its subsequent disease potential. In phosphate-rich pigeon guano, fungal cells tend to develop into small capsules that tend to disseminate into the liver and spleen but with moderate dissemination to the CNS. In contrast, exposure to inositol-rich eucalyptus environments induces small-cell, large-capsule morphotypes that have cell wall differences are optimized for blood-brain barrier traversal and CNS invasion. The model proposes that environmental nutrient pre-conditioning contributes to regional differences in disease severity, particularly in sub-Saharan Africa and Asia where eucalyptus exposure is common.

This opinion piece aims to reframe our understanding of CM by emphasizing the ecological context in which *C. neoformans* develops virulent traits (**Figure 2**). While disease outcome has usually been linked to host and pathogen factors, new research shows that *C. neoformans’* life outside the host also plays a role, especially environmental nutrient landscapes (pigeon guano and Eucalyptus trees) may play a pivotal role in priming fungal phenotypes for CNS invasion (Barbosa and Schultz, 1987; Hallas-Møller et al., 2024). We argue that understanding how *C. neoformans* adapts to its environmental niche, is critical to deciphering the ecological drivers of its virulence and the geographic burden of cryptococcal disease and ultimately mitigate the global disparities in cryptococcal disease outcomes.

## From droppings to disease: phosphate- and urea-rich pigeon guano as an ecological driver of *C. neoformans* dissemination and virulence

Pigeon guano, common in urban environments, is more than a passive reservoir for *C. neoformans*, it is a nutrient-rich ecological niche capable of actively shaping fungal virulence. Rich in nitrogen, phosphorus, and potassium, guano provides an ideal substrate for fungal proliferation, mating, and spore production (Nielsen et al., 2007; Duffaut et al., 2023).

### Phosphate as a dissemination cue

Among these nutrients, phosphate is both a critical growth resource and a potent signalling molecule. *In vitro* culture of *C. neoformans* in phosphate-rich conditions generate small-cell, thin-capsule morphotypes identical to those observed in guano-grown strains (Denham et al., 2022). These phenotypes enhance macrophage uptake and promote extrapulmonary dissemination to organs such as the spleen and liver (Denham et al., 2022). Beyond its role in nucleic acid synthesis, ATP production, and membrane construction, phosphate abundance can activate fungal phosphate-sensing pathways (e.g., the PHO regulon) (Denham et al., 2022), reprogramming metabolism toward traits that improve persistence and systemic spread. Similar phosphate-linked modulation of pathogenic traits occurs in *Candida albicans* (enhanced adhesion and biofilm formation), *Pseudomonas aeruginosa* (nutrient abundance supporting persistence in sputum), and *Mycobacterium tuberculosis* (upregulated phosphate transporters aiding intracellular survival) (Chekabab et al., 2014; Khan et al., 2024).

### Urea as a CNS facilitator

Nitrogenous compounds, particularly urea, add a second dimension to guano’s virulence potential. Urea induces the expression of urease, a well-characterized virulence factor that facilitates tissue invasion, immune modulation (Denham et al., 2022), and traversal across host barriers (Burne and Chen, 2000; Cox et al., 2000; Kusters et al., 2006; Singh et al., 2013). Deletion of the urease gene in *C. neoformans* significantly impairs CNS invasion in experimental models (Cox et al., 2000; Olszewski et al., 2004). While guano-associated phosphate morphotypes appear more tuned for systemic dissemination than for neurotropism, the co-occurrence of urea could increase CNS risk under specific host conditions (Olszewski et al., 2004). For instance, elevated circulating urea, seen in renal impairment (Vanholder et al., 2018), severe dehydration (Hayajneh et al., 2010), advanced HIV infection (Olusola et al., 2020), or prolonged critical illness (Zijlstra et al., 2024), may create a physiological context in which guano-primed strains gain enhanced access to the brain. Overall, pigeon guano acts as an ecological filter that selects for dissemination-competent phenotypes with moderate CNS-invasive potential, but still clinically relevant when host metabolic conditions amplify urease-dependent pathways.

## From bark to brain: how inositol in eucalyptus trees primes *Cryptococcus* for brain infection

Eucalyptus trees, particularly *Eucalyptus camaldulensis*, are widely distributed in regions with high burdens of cryptococcal meningitis (CM) (Dessie and Erkossa, 2011; Rajasingham et al., 2017) and serve as established environmental reservoirs for both *C. neoformans* and *C. gattii* (Granados and Castañeda, 2005; Refojo et al., 2009; Girish Kumar et al., 2010; Elhariri et al., 2016; May et al., 2016; Edwards et al., 2021). These trees are enriched in inositol (Souden et al., 2020), a sugar alcohol now recognized as a potent regulator of cryptococcal neurotropism (Liu et al., 2013). Unlike most fungi, which have only one or two inositol transporter (ITR) genes, *C. neoformans* possesses a markedly expanded inositol transporter (ITR) gene family encoding 10–11 transporters, reflecting a strong evolutionary adaptation to inositol-rich habitats (Xue et al., 2010). In line with this, Population genomic analyses by Desjardins et al. (2017) revealed that inositol transporters and inositol catabolism genes are under strong positive selection across all major VN lineages of *C. neoformans* (Desjardins et al., 2017). These findings indicate that inositol acquisition pathways have been repeatedly shaped by evolutionary pressure, supporting long-term adaptation to inositol-rich niches such as Eucalyptus trees, and ultimately the human brain, where inositol is abundant.

### Inositol as a CNS-tropic signal

High environmental inositol induces small-cell morphotypes with thicker capsules, phenotypes that mirror those found in brain-isolated cryptococcal cells and are strongly linked to enhanced dissemination to CNS and survival (Wang et al., 2021). Inositol not only drives capsule biosynthesis but also promotes dose-dependent traversal across blood-brain barrier monolayers (Liu et al., 2013). This is biologically significant because brain inositol concentrations are 50-100x higher than in plasma (Fisher et al., 2002), and levels are often further elevated in people living with HIV (Chang et al., 2004), creating a nutrient gradient that actively draws inositol-primed strains toward the CNS.

### Pre-conditioning and “molecular memory”

Memory of past events can shape the adaptation of a pathogen and lead to better survival inside the hosts and increase damage to the host. In this regard *C. neoformans* yeast or spores primed by the environment can rapidly re-express CNS-tropic traits through transcriptional or epigenetic memory (Meriem et al., 2019; Harish and Osherov, 2022). In murine models, *C. neoformans* mutants defective in inositol metabolism fail to penetrate the CNS, form abnormally large cells, and remain largely confined to the lungs (Wang et al., 2011; Wang et al., 2021). Additionnally cells that are precultured in inositol containing media displayed increase in CNS dissemination and capsule enlargement in capsule inducing media (Wang et al., 2021). Inositol fuels synthesis of UDP-glucuronic acid, a precursor for both the polysaccharide capsule and hyaluronic acid, the latter enabling blood-brain barrier crossing via host CD44 receptor engagement (Liu et al., 2013; Wang et al., 2021). The interaction of *Cryptococcus neoformans* with Eucalyptus trees in the environment serves not only as an ecological niche but also as a source of bioactive compounds such as high concentrations of inositol that can precondition the fungus for brain invasion in human hosts. This environmental priming may partly explain regional differences in the severity of cryptococcal meningitis among HIV-infected patients exposed to *C. neoformans* from distinct ecological sources.

### Comparative perspective

Nutrient priming of virulence is not unique to fungi. For instance, in the fish bacterial pathogen *Flavobacterium columnare*, growth in double concentration of Shieh medium significantly upregulates the expression of key virulence determinants, including the chondroitinase gene (*cslA*) and collagenase genes, compared with cells grown in the standard or low concentration (Penttinen et al., 2016). This elevated expression correlates with a more rapid onset of disease and higher mortality in infected fish (Penttinen et al., 2016). The upregulation of virulence factors that are observed may be due to linkage between nutrient sensing and global metabolic regulation systems such as carbon catabolite repression, nitrogen phosphotransferase system, stringent response pathways (ppGpp) mediated regulation which integrates environmental nutrient cues with virulence genes expressions (Görke and Stülke, 2008; Dalebroux et al., 2010; Pflüger-Grau and Görke, 2010). In general, this reflects a broader principle: nutrient-sensing pathways can be wired directly into virulence regulation, enabling pathogens to translate environmental abundance into host-adaptive traits. Together, Inositol-rich Eucalyptus niches appear to be powerful ecological incubators for CNS-tropic *C. neoformans* phenotypes, driving brain invasion more efficiently and predictably than guano-associated strains. This could partially explain why CM outcomes are often more severe and rapid in eucalyptus-rich regions, even when anti-fungal therapy is available.

## Conclusion

Regional disparities in cryptococcal meningitis outcomes cannot be fully explained by host immunity or healthcare access alone. Evidence increasingly supports a nutrient-priming model of virulence, in which environmental nutrient profiles pre-condition *C. neoformans* for specific pathogenic trajectories (Liu et al., 2013; Wang et al., 2021). Pigeon guano, rich in phosphate and urea, selects for small-cell, thin-capsule morphotypes optimized for systemic dissemination, with only moderate CNS-invasive potential except under certain host conditions such as hyperuremia. In contrast, inositol-rich eucalyptus habitats appear to act as ecological incubators for highly CNS-tropic phenotypes, driving rapid and severe neuroinvasion. This highlights that C. neoformans strains differ significantly in their capacity for immediate CNS invasion. Consequently, the development of cryptococcal meningitis should not be regarded as an inevitable outcome of exposure but rather as a risk influenced by a complex interplay of environmental conditions, pathogen variability, and host physiology. This framework underscores the importance of considering both ecological and biological factors in understanding disease progression. What we have proposed represents associations observed in the currently available data. Direct testing of this model will require rigorous comparative epidemiological studies across regions with distinct environmental reservoir profiles, supported by integrated surveillance of nutrient availability, climatic conditions, and other ecological determinants that may modulate pathogen virulence and host susceptibility. Such work could inform predictive mapping of cryptococcal meningitis risk and interventions that disrupt nutrient-responsive virulence pathways before they manifest clinically. In sum, this nutrient-centric model of cryptococcal pathogenesis has critical implications for public health:

1. Environmental surveillance should expand beyond urban pigeon guano to include systematic sampling of eucalyptus habitats near human dwellings, particularly in endemic regions.
2. Disease prediction and environmental risk-mapping efforts may benefit from also quantifying inositol levels in additional tree species known to harbor Cryptococcus neoformans, such as mopane (Colophospermum mopane) and olive trees. Incorporating these data into ecological surveillance models could help identify regions with increased environmental suitability for CNS-pre-conditioned Cryptococcus and support targeted public awareness and preventative health messaging.
3. Intervention strategies may include environmental management approaches that reduce fungal load in high-risk ecological niches, as well as the development of new anti-fungal drugs that target nutrient-responsive virulence pathways. Such strategies would expand the current anti-fungal arsenal and offer additional means to prevent or control infection in regions where environmental exposure risk is elevated.

## Data Availability

The original contributions presented in the study are included in the article/**Supplementary Material**. Further inquiries can be directed to the corresponding authors.
