# Correction: The emergence of neuroinvasive Cryptococcus: why eucalyptus-rich regions, especially in Africa, may be facing greater risk

**DOI:** 10.3389/fcimb.2026.1868340

**Published:** 2026-06-15

**Authors:** Hilaire Irere, Rachael Dangarembizi, Liliane Mukaremera, Ivy M. Dambuza

**Affiliations:** 1 Medical Research Council Centre for Medical Mycology at the University of Exeter, Department of Biosciences, Faculty of Health and Life Sciences, Exeter, United Kingdom; 2 Division of Physiological Sciences, Department of Human Biology, Faculty of Health Sciences, University of Cape Town, Cape Town, South Africa; 3 Neuroscience Institute, Faculty of Health Sciences, University of Cape Town, Groote Schuur Hospital, Cape Town, South Africa; 4 Medical Research Council Centre for Medical Mycology Africa Unit, Cape Town, South Africa

**Keywords:** Cryptococcus neoformans, eucalyptus, inositol, neurotropism, virulence

In the published article, an incorrect citation was used in the section “From bark to brain: how inositol in eucalyptus trees primes Cryptococcus for brain infection,” subsection “*Inositol as a CNS-tropic signal*”. The sentence:

“…levels are often further elevated in people living with HIV (Bertran-Cobo et al., 2022)…”

incorrectly cited “Bertran-Cobo et al. (2022)”. That study examined neurometabolite profiles, including myo-inositol, in children who were HIV-exposed but uninfected — not in individuals living with HIV. This citation therefore does not support the stated claim.

The citation has been replaced with “Chang et al. (2004)”, a multicenter *in vivo* proton magnetic resonance spectroscopy (MRS) study that directly demonstrated significantly elevated myoinositol-to-creatine ratios (MI/Cr) in HIV-positive patients, both in neuroasymptomatic individuals and those with AIDS dementia complex, compared to HIV-seronegative controls. The corrected sentence reads:

“…levels are often further elevated in people living with HIV (Chang et al., 2004)…”

In the **Reference** list, the **Reference** for “Bertran-Cobo et al. (2022)” has been removed and replaced with the following:

“Chang L, Lee PL, Yiannoutsos CT, Ernst T, Marra CM, Richards T, Kolson D, Schifitto G, Jarvik JG, Miller EN, Lenkinski R, Gonzalez G and Navia BA (2004) A multicenter *in vivo* proton-MRS study of HIV-associated dementia and its relationship to age. NeuroImage 23:1336–1347. doi: 10.1016/j.neuroimage.2004.07.067”

The original version of this article has been updated.

